# Whole-blood methylation signatures are associated with and accurately classify multiple sclerosis disease severity

**DOI:** 10.1186/s13148-022-01397-2

**Published:** 2022-12-30

**Authors:** Maria Pia Campagna, Alexandre Xavier, Rodney A. Lea, Jim Stankovich, Vicki E. Maltby, Helmut Butzkueven, Jeannette Lechner-Scott, Rodney J. Scott, Vilija G. Jokubaitis

**Affiliations:** 1 Central Clinical School, Monash University, Melbourne, VIC Australia; 2 Monash University, Melbourne, VIC Australia; 3 Hunter Medical Research Institute, University of Newcastle, Newcastle, NSW Australia; 4 Queensland University of Technology, Brisbane, QLD Australia; 5 University of Melbourne, Melbourne, VIC Australia; 6 Royal Melbourne Hospital, Melbourne, VIC Australia; 7 Neurology Department, Eastern Health, Melbourne, VIC Australia; 8 Neurology Department, Alfred Health, Melbourne, VIC Australia; 9 Neurology Department, John Hunter Hospital, Hunter New England Health, Newcastle, NSW Australia; 10 School of Biomedical Sciences and Pharmacy, University of Newcastle, Newcastle, NSW Australia; 11 Division of Molecular Medicine, New South Wales Health Pathology North, Newcastle, NSW Australia

**Keywords:** DNA methylation, Epigenetics, Prognostics, Machine learning, Epigenome-wide association study, Genomics

## Abstract

**Background:**

The variation in multiple sclerosis (MS) disease severity is incompletely explained by genetics, suggesting genetic and environmental interactions are involved. Moreover, the lack of prognostic biomarkers makes it difficult for clinicians to optimise care. DNA methylation is one epigenetic mechanism by which gene–environment interactions can be assessed. Here, we aimed to identify DNA methylation patterns associated with mild and severe relapse-onset MS (RMS) and to test the utility of methylation as a predictive biomarker.

**Methods:**

We conducted an epigenome-wide association study between 235 females with mild (*n* = 119) or severe (*n* = 116) with RMS. Methylation was measured with the Illumina methylationEPIC array and analysed using logistic regression. To generate hypotheses about the functional consequence of differential methylation, we conducted gene set enrichment analysis using *ToppGene*. We compared the accuracy of three machine learning models in classifying disease severity: (1) clinical data available at baseline (age at onset and first symptoms) built using elastic net (EN) regression, (2) methylation data using EN regression and (3) a weighted methylation risk score of differentially methylated positions (DMPs) from the main analysis using logistic regression. We used a conservative 70:30 test:train split for classification modelling. A false discovery rate threshold of 0.05 was used to assess statistical significance.

**Results:**

Females with mild or severe RMS had 1472 DMPs in whole blood (839 hypermethylated, 633 hypomethylated in the severe group). Differential methylation was enriched in genes related to neuronal cellular compartments and processes, and B-cell receptor signalling. Whole-blood methylation levels at 1708 correlated CpG sites classified disease severity more accurately (machine learning model 2, AUC = 0.91) than clinical data (model 1, AUC = 0.74) or the wMRS (model 3, AUC = 0.77). Of the 1708 selected CpGs, 100 overlapped with DMPs from the main analysis at the gene level. These overlapping genes were enriched in neuron projection and dendrite extension, lending support to our finding that neuronal processes, rather than immune processes, are implicated in disease severity.

**Conclusion:**

RMS disease severity is associated with whole-blood methylation at genes related to neuronal structure and function. Moreover, correlated whole-blood methylation patterns can assign disease severity in females with RMS more accurately than clinical data available at diagnosis.

**Supplementary Information:**

The online version contains supplementary material available at 10.1186/s13148-022-01397-2.

## Background

Multiple sclerosis (MS) is an autoimmune, neurodegenerative disease that is highly heterogeneous between individuals. Variability in disease activity and trajectory is demonstrated in natural history as well as modern day cohorts, despite disease-modifying therapy (DMT) exposure [1]. Clinicians are currently unable to predict patients’ future disease severity due to a lack of prognostic biomarkers, and as such, selecting the most appropriate course of management for the individual remains a clinical challenge.

Genome-wide association studies (GWASes) have identified over 200 independent MS susceptibility variants [2]. Therefore, it is logical that heterogeneity of MS clinical outcomes could also be regulated by genetic factors. However, attempts to associate MS susceptibility variants with disease activity [3] or disease progression [4] have been largely unsuccessful. Similarly, discovery GWASes of disease severity using cross-sectional MS severity scale scores have also failed to identify any variants reaching genome-wide significance [5, 6]. Epidemiological studies demonstrate the impact of environmental factors on disease severity, most notably demonstrating that smoking [7] and vitamin D deficiency [8] are associated with an increased risk of more severe disease, while DMT exposure and pregnancy with a reduced risk [9]. This points us to consider epigenetic mechanisms as the missing link explaining the observed clinical heterogeneity in disease severity.

DNA methylation is the best understood epigenetic mechanism that involves the presence or absence of a methyl (CH_3_) group on cytosine–phosphate–guanine (CpG) dinucleotides in the DNA sequence. Importantly, methylation is impacted by environmental factors and, therefore, is a plausible mechanism by which genetic and environmental factors interact to drive disease susceptibility and severity.

In case–control epigenome-wide association studies (EWASes) of MS, differential methylation in the HLA gene is the strongest validated finding across studies and cell types [10–13]. EWASes comparing methylation patterns between MS subtypes are less common, therefore methylation signatures specific to relapsing–remitting MS (RRMS), secondary progressive MS (SPMS) or primary progressive MS (PPMS) are not validated. PPMS patients show general hypermethylation in whole blood compared to RRMS patients [14], while RRMS and SPMS patients exhibit differences in CD4+ [12, 15–17] and CD8+ T cell [17], CD14+ monocyte[17] and CD19+ B cell [17] methylation patterns. In CD4+ T cells, differential methylation at the Major Histocompatibility Complex (including HLA-DRB1 and HLA-DRB5) [12], hypomethylation of the HTR2A locus [16] and hypermethylation at the VMP1/MIR21 locus [15] in SPMS compared to RRMS has been shown. One study has demonstrated SPMS-specific signatures in CD8+ T cells, CD14+ monocytes and CD19+ B cells compared to RRMS and controls [17]. These signatures were enriched in neuronal and neurodegenerative genes, and myeloid cell function [17]. Despite these comparisons of MS subtypes, no studies to date have compared the methylation patterns between people at the extremes of relapse-onset MS (RMS) severity; where RMS refers to patients diagnosed with RRMS and living with either RRMS or SPMS at blood collection.

In this study, we aimed to identify whole-blood and immune cell-specific DNA methylation patterns associated with mild and severe RMS across autosomes. Additionally, we aimed to assess differences in methylation age acceleration between groups and further to test the utility of DNA methylation as a predictive biomarker in MS compared to clinical data available at diagnosis.

## Results

### Cohort characteristics

This study included 235 females with relapse-onset MS across four study sites (Additional file 1: Fig. S1). Using MSBase Registry data, we determined disease severity using a longitudinal age-related multiple sclerosis severity (ARMSS) score [18] approach. For each patient, an ARMSS score was calculated for each visit with a relapse-independent Expanded Disability Status Scale (EDSS) [19] score recorded. The median of these ARMSS scores was the longitudinal ARMSS score used to classify severity. Patients with median longitudinal ARMSS scores at or below the cohort 20th percentile were categorised as mild, while those at or above the 80th percentile were categorised as severe. Mild and severe patients had median longitudinal ARMSS scores of 1.21 (range = 0.17–3.01) and 8.36 (range = 6.52–9.92), respectively (Table 1).

**Table 1:** Cohort summary statistics

Characteristics		Mild (*n* = 119)	Severe (*n* = 116)	All (*n* = 235)	Cohen’s d
Longitudinal ARMSS score	Median (IQR)	1.21 (0.72, 1.86)	8.63 (7.86, 9.20)	2.86 (1.20, 8.62)	8.87
	Range	0.17–3.01	6.52–9.92	0.17–9.92	
Disease course at blood collection	RRMS	118 (99.2%)	46 (39.6%)	163 (69.4%)	–
	SPMS	0 (0.0%)	70 (60.4%)	71 (30.6%)	
Disease course at most recent visit	RRMS	117 (98.3%)	39 (33.6%)	157 (66.8%)	
	SPMS	1 (0.008%)	77 (66.4%)	78 (33.2%)	
Sex	Female	119 (100.0%)	116 (100.0%)	235 (100.0%)	–
	Male	0 (0%)	0 (0%)	0 (0%)	
Age at most recent visit	Median (IQR)	53.57 (45.92, 62.14)	53.47 (45.34, 61.33)	52.41 (43.81, 59.31)	0.15
	Range	29.00–76.35	28.80–76.35	28.80–76.21	
Age at blood collection	Median (IQR)	49.10 (40.75, 57.62)	48.40 (40.10, 56.50)	47.80 (39.85, 55.35)	0.11
	Range	24.10–72.20	24.00–73.00	24.00–73.00	
Symptom duration (years)	Median (IQR)	15.90 (11.45, 20.98)	24.18 (19.65, 31.70)	19.92 (13.79, 26.49)	0.95
	Range	5.85–38.11	11.91–47.13	5.85–47.13	
ARR	Median (IQR)	0.10 (0.00, 0.20)	0.17 (0.00, 0.38)	0.11 (0.00, 0.26)	0.39
	Range	0.00–0.82	0.00–1.08	0.00–1.08	
DMT at blood collection	Yes	84 (70.5%)	68 (58.6%)	152 (64.7%)	–
	No	35 (29.5%)	48 (41.4%)	83 (35.3%)	
Follow-up in MSBase (years)	Median (IQR)	11.76 (10.24, 14.24)	11.27 (9.78, 13.29)	11.13 (9.49, 12.59)	0.25
	Range	5.15–21.96	5.15–32.16	5.26–32.16	
Number of EDSS scores assessed	Median (IQR)	15.00 (11.00, 21.25)	15.00 (11.00, 19.00)	14.00 (10.00, 18.00)	0.29
	Range	3.00–47.00	3.00–47.00	3.00–34.00	

*ARMSS* age-related multiple sclerosis severity score, *IQR* interquartile range, *EDSS* expanded disability status scale, *ARR *annualised relapse rate, *DMT* disease-modifying therapy

*MS course data missing for one patient

### Disease severity is associated widespread differential methylation in whole-blood

After methylation data preprocessing using the *Chip Analysis Methylation Pipeline (ChAMP)* Bioconductor package [20], approximately 748,000 of 867,000 (86%) probes remained for analysis (Additional file 1: Fig. S2). Batch effect analysis identified Plate, Sentrix ID and Sentrix Position as significant sources of technical variation (*p* < 0.01). Batch effect correction reduced these to negligible effects (Additional file :1 Fig. S3).

**Fig. 1:**
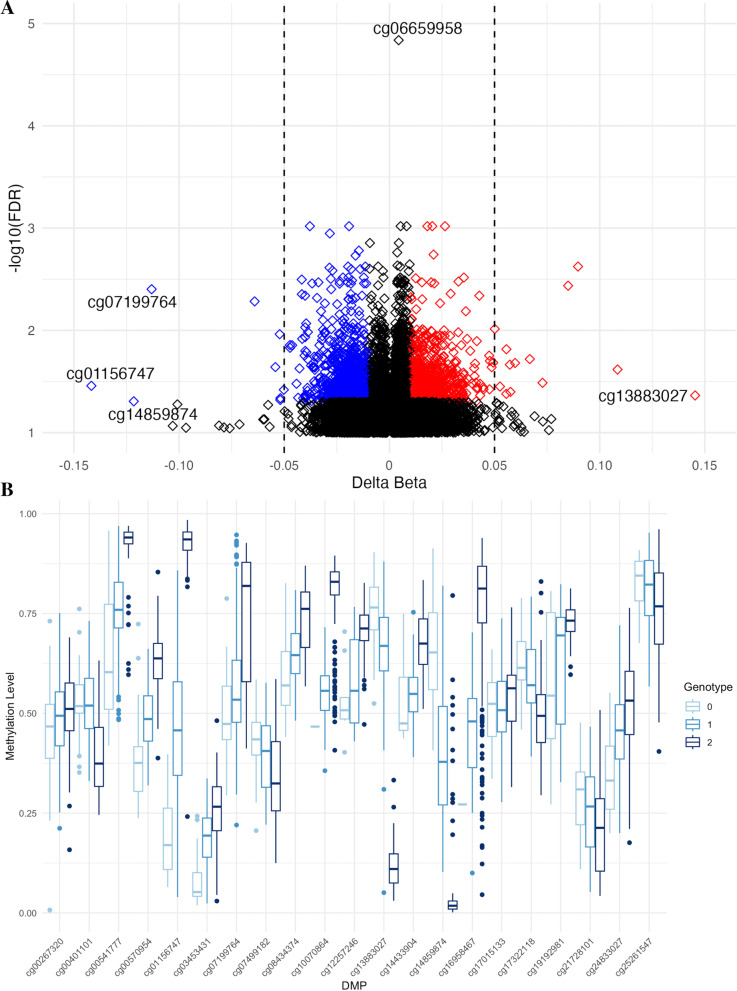
Differentially methylated positions (DMPs) between mild and severe groups in whole-blood.** A** Of 1472 DMPs, 839 (57%) were hypomethylated (shown in blue) and 633 (43%) were hypermethylated (shown in red) in the severe group. 22 DMPs had a Δ_meth_ above 5% (indicated by the dashed line). **B** Methylation quantitative trait loci (mQTLs) for each major DMP (Δ_meth > 5%)._ Methylation beta value for each DMP is plotted by genotype at the most significant mQTL based on p-value. The plotted CpG-SNV pairs are: cg10070864-15:57665782 (*p* = 7.25 × 10^–15^), cg07199764-1:5348270 (*p* = 7.50 × 10^–13^), cg07499182-13:33824770 (*p* = 4.76 × 10^–06^), cg17015133-1:155106697 (*p* = 4.17 × 10^–02^), cg14433904-8:78519337 (*p* = 4.33 × 10^–12^), cg24833027-8:1902112 (*p* = 1.43 × 10^–10^), cg00401101-5:16508674 (*p* = 1.01 × 10^–17^), cg08434374-11:44471561 (*p* = 5.46 × 10^–05^), cg03453431-7:157226016 (*p* = 7.05 × 10^–21^), cg16958467-15:57655776 (*p* = 8.69 × 10^–29^), cg00541777-2:3652616 (*p* = 5.22 × 10^–28^), cg01156747-7:120429 (*p* = 9.31 × 10^–36^), cg17322118-1:85744472 (*p* = 2.14E-12), cg12257246-8:25240100 (*p* = 4.73E-17), cg00267320-10:111537595 (*p* = 3.73E-02), cg00570954-12:4281245 (*p* = 2.50 × 10^–26^), cg13883027-2:62887884 (*p* = 3.11 × 10^–38^), cg25261547-19:49364511 (*p* = 7.81 × 10^–03^), cg21728101-7:1976457 (*p* = 3.48 × 10^–04^), cg14859874-1:154239283 (*p* = 1.13 × 10^–30^), cg19192981-15:57667027 (*p* = 2.28 × 10^–06^). The list of mQTLs and p-values for each major DMP are listed in Additional file 2: Table S12. Δ_meth_ delta beta, SNV single-nucleotide variant

To identify differential methylation between mild and severe groups at the single CpG level [i.e. differentially methylated positions (DMPs)], we implemented a logistic model of methylation level at each probe and severity group, adjusted for Natural Killer (NK) cell proportions. NK cell proportions were significantly associated with 1514 CpGs (FDR < 0.05, Additional file :2 Table S1) in sensitivity analyses and were therefore adjusted for in the differential methylation analysis. We revealed 1472 DMPs with an FDR < 0.05 and methylation difference (Δ_meth_) > 1% between mild and severe groups, mapping to 812 genes and 660 unannotated genomic locations (major DMPS listed in Table 2, full list shown in Additional file 2: Table S2). Of these 1472 DMPs, 839 (57%) were hypomethylated and 633 (43%) were hypermethylated in the severe group relative to the mild group (Fig. 1A). Δ_meth_ ranged from -14.02 to 14.04%. The majority of DMPs were in open sea regions (1039, 70.6%), with 264 (17.9%) in shores, 105 (7.1%) in shelves and 64 (4.3%) in islands.

**Table 2:** Major differentially methylated positions (DMPs, Δmeth > 5%)

DMP	Chr	BP	Gene	Feature	CGI	Δmeth	FDR	mQTLs (GRCh37*)*
cg10070864	15	57664490		IGR	Shelf	0.090	0.002	15:57665782 15:57667027
cg07199764	1	5347468		IGR	Opensea	− 0.113	0.004	1:5343656 1:5346474 1:5346800 1:5347155 1:5347457 1:5348270 1:5348478 1:5350257
cg19192981	15	57664704		IGR	Shelf	0.085	0.004	15:57665782 15:57667027
cg07499182	13	33825496	STARD13	Body	Opensea	− 0.064	0.005	13:33824770 13:33824988 13.33826125 13.33829245 13:33829571
cg25304129	22	26125490		IGR	Opensea	0.050	0.010	–
cg17015133	1	155111332	RAG1AP1	3'UTR	Shore	− 0.052	0.011	1:155106697
cg14433904	8	78518428		IGR	Opensea	0.056	0.015	8:78519059 8.78519337
cg24833027	8	1897969	ARHGEF10	Body	Shelf	0.067	0.019	8:1902750 8:1899691 8:1902112 8:1897657 8:1900410 8:1900911 8:1900546 8.1901176 8.1899381 8:1895934
cg00401101	5	16509323	FAM134B	TSS1500	Opensea	0.060	0.021	5:16508674 5:16512364 5:16514269
cg08434374	11	44467306		IGR	Opensea	0.057	0.022	11:44469734 11:44471561
cg03453431	7	157225567		IGR	Opensea	− 0.054	0.023	7:157226016
cg16958467	15	57655799		IGR	Opensea	0.108	0.024	15:57655776 15:57656919
cg00541777	2	3652840	COLEC11	TSS1500	Opensea	0.073	0.033	2:3650466 2:3652616 2:3654290 2:3654683
cg01156747	7	120659		IGR	Island	− 0.142	0.035	7:120429 7:120174 7:124276 7:124586 7:116077 7:116054 7:116969 7:122450 7:121755 7:117441
cg17322118	1	85743732	BCL10	TSS200	Shore	0.053	0.035	1:85744472 1:85740024 1:85742860 1:85738953
cg12257246	8	25240570	DOCK5	Body	Opensea	− 0.050	0.038	8:25240100 8:25245432
cg00267320	10	111533258		IGR	Opensea	0.058	0.040	10:111528480 10:111537595
cg00570954	12	94281309		IGR	Opensea	0.056	0.042	12:94281245 12:94285952 12:94282740
cg13883027	2	62892060		IGR	Opensea	0.145	0.043	2:62887884 2:62890171 2:62892323
cg25261547	19	49363369	PLEKHA4	Body	Opensea	− 0.052	0.046	19:49364511
cg21728101	7	1979360	MAD1L1	Body	Shore	− 0.052	0.048	7:1976457 7.1983928
cg14859874	1	154238265	UBAP2L	Body	Opensea	− 0.122	0.049	1:154238063 1:154239283 1:154243115 1:154243245

*Δmeth* delta methylation beta value; *FDR* false discovery rate; *CHR* chromosome; *BP* base position, *CGI* CpG island, *mQTL* methylation quantitative trait loci, *IGR* intergenic region, *TSS200* transcription start site 200, *TSS1500* transcription start site 1500, *3*′*UTR* 3′ untranslated region

No differentially methylated regions (DMRs) were identified using the *DMRcate* algorithm at an FDR threshold of 0.05. Therefore, we loosened the definition of a DMR to a region containing at least two DMPs with the same effect direction, within 1000 bp of the adjacent DMP/s and using an FDR < 0.01. With this definition, suggestive DMRs on chromosomes 11 and 15 were identified containing two DMPs each (hereafter referred to DMR^Chr11^ and DMR^Chr15^, **Table ****3**). DMR^Chr11^ was hypomethylated in the severe group with a maximum Δ_meth_ of 1.8% (Additional file 1: Fig. S4A). The strongest DMP in this DMR was cg02877698 (Δ_meth_ = 1.8%, FDR = 0.0087). DMR^Chr11^ contained two DMPs within a 447-bp region that overlapped with the gene body of uncharacterised long noncoding RNA *LOC101929295*. DMR^Chr15^ was hypermethylated in the severe group with a maximum Δ_meth_ of 8.7% (Additional file 1: Fig. S4B). cg10070864 was the strongest DMP in this DMR (Δ_meth_ = 8.7%, FDR = 0.0032). DMR^Chr15^ contained two DMPs within a 214-bp unannotated, intergenic region.

**Table 3:** Differentially methylated regions (DMRs)

CHR	Start (bp)	End (bp)	Width	n CpGs	Max Δ_meth_	Mean Δ_meth_	Overlapping genes	Feature	CGI	CpGs	mQTLs (GRCh37*)*
11	94886261	94886708	447	2	− 0.018	− 0.018	LOC101929295	IGR, Body	shelf	cg10357314, cg02877698	11:94886463 11:94886632
15	57664490	57664704	214	2	0.087	0.085	NA	IGR	shore	cg10070864, cg19192981	15:57664572

*CHR* chromosome, *bp* base pair, *CpG* cytosine–phosphate–guanine, *diff* difference, *CGI* CpG Island, *max* maximum, *mQTL* methylation quantitative trait loci

### Disease severity is associated with CD8+ T cell methylation

**Fig. 2:**
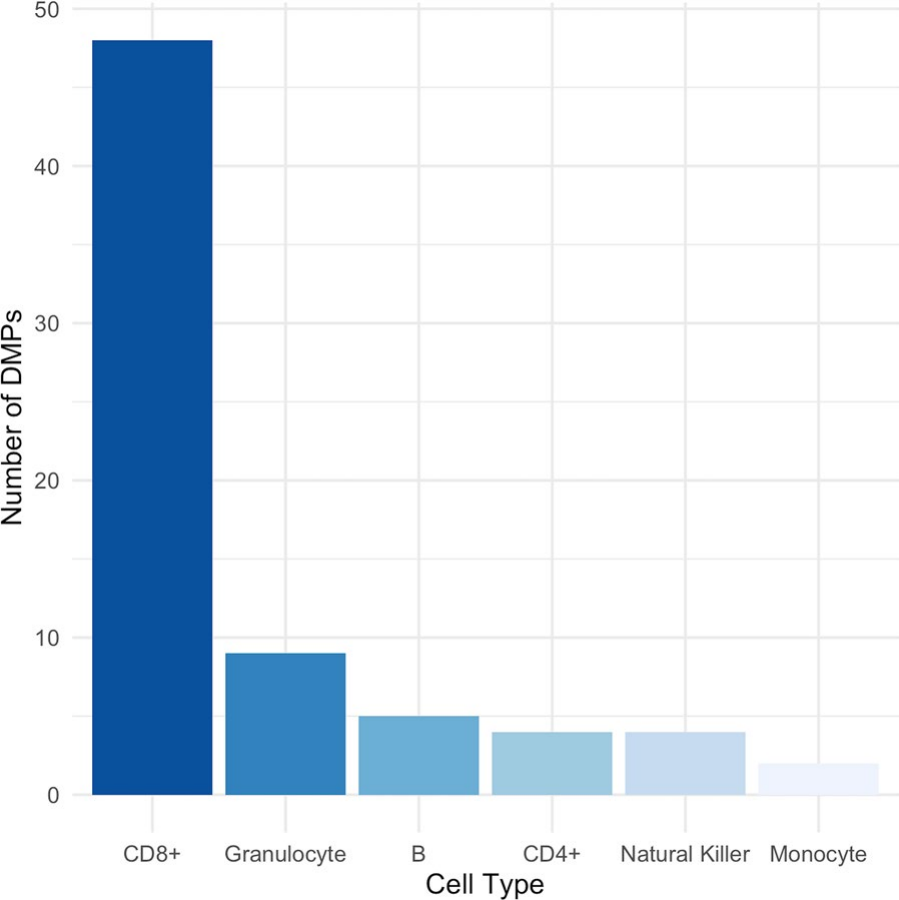
Number of differentially methylated positions (DMPs) in each immune cell type. CD8 + T cells (*n* = 48), granulocytes (*n* = 8), B cells (*n* = 5), CD4 + T cells (*n* = 4), Natural Killer cells (*n* = 4), monocytes (*n* = 2)

As methylation can be cell-type-specific, differential methylation analysis of whole-blood signal may not be sensitive to cell-specific DMPs (csDMPs). To address this, we estimated and compared the proportion of immune cell types in mild and severe groups. Cell-type proportions were estimated from whole-blood data using reference-based statistical deconvolution with the CIBERSORT algorithm [21]. No differences were found in immune cell proportions (Additional file 1: Fig. S5). We identified csDMPs in all cell types tested using linear models (Fig. 2): 48 csDMPs in CD8+ T cells, eight in granulocytes, five in B cells**,** four in CD4+ T cells, four in NK cells and two in monocytes (Additional file :2 Tables S3–8).

### Natural Killer cell proportions are associated with methylation patterns

In sensitivity analyses, NK cell proportions were significantly associated with 1,514 CpGs (FDR < 0.05, Additional file :2 Table S1). No CpGs were significantly associated with other covariates tested (data not shown), demonstrating no major effects of these covariates on differential methylation in our cohort and therefore were not adjusted for in the analysis. Of the 2622 smoking-associated CpGs form Joehanes (2016) and 1472 DMPs identified in this study, 127 overlapped and were removed prior to downstream analyses to avoid confounding (Additional file 2: Table S9).

### Disease severity is associated with methylation independent of genetic effects

Methylation quantitative trait loci (mQTLs) refer to single-nucleotide variants (SNVs) that influence methylation levels at or near certain genetic loci. We conducted a targeted mQTL analysis by testing the relationship between genotype and methylation levels within a) a ± 5 kb window of each major DMP (Δ_meth_ > 5%) and b) at each DMR.

There were 72 independent SNVs across the 22 major DMPs (Table 2**, **Fig. 1B). After quality control and filtering, 221 of 235 samples had genotype data for analysis. All major DMPs except cg25304129 contained at least one mQTL (Additional file 2: Table S10). We adjusted our differential methylation analysis for mQTL effects by modelling methylation and genotype as joint predictors of disease severity in a logistic model. The association between methylation level and disease severity at these major DMPs was not attenuated when adjusted for genotype (Additional file 2: Table S10).

Three independent SNPs in DMR^Chr11^ and one independent SNP in DMR^Chr15^ were assessed for mQTL effects. In DMR^Chr11^, genotype at 11:94,886,463 and 11:94,886,632, but not 11:94,886,601, were correlated with methylation, suggesting mQTL effects at this DMR (Additional file 1: Fig. S6, Additional file 2: Table S11). Adjusted logistic models showed that DMR^Chr11^ remains significantly associated with disease severity, independent of genotype at 11:94,886,463 (cg10357314, *p* = 1.61 × 10^–05^ and cg02877698, *p* = 2.15 × 10^–06^) and 11:94,886,632 (cg10357314, *p* = 0.00017 and cg02877698, *p* = 4.72 × 10^–06^). In DMR^Chr15^, genotype at 15:57,664,572 was correlated with methylation (Additional file 1: **Fig. S7,** Additional file 2: **Table S11**). Accounting for 15:57,664,572 genotype in a logistic model demonstrated that DMR^Chr15^ remains significantly associated with disease severity (cg10070864, *p* = 4.06 × 10^–29^ and cg19192981, *p* = 4.20 × 10^–29^).

### Disease severity is associated with differential methylation at genes enriched in neuronal compartments and pathways

**Fig. 3:**
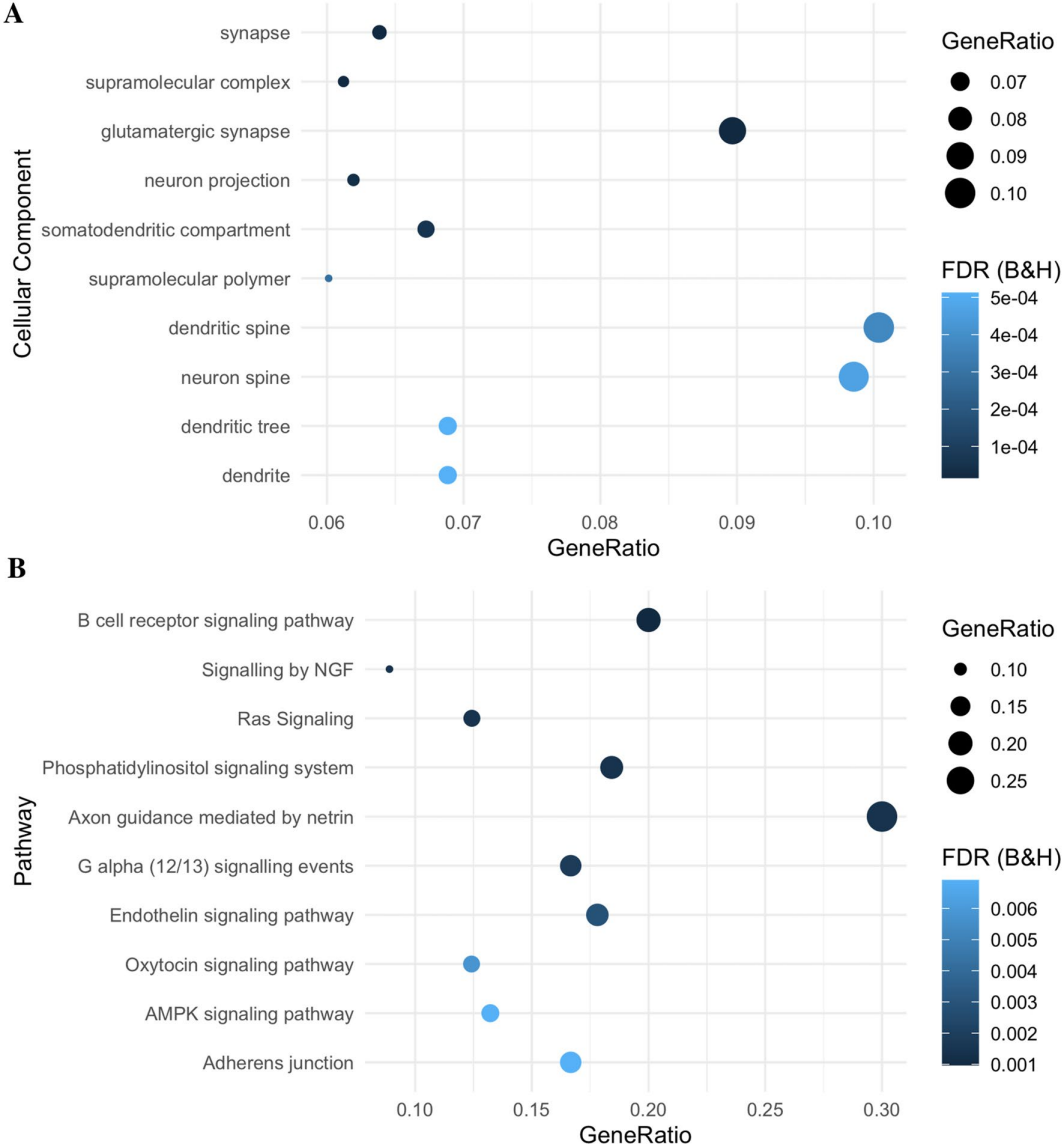
Gene Set Enrichment Analysis (GSEA) of differentially methylated positions (DMPs). GSEA was conducted using 812 genes. **A** Ten most significantly enriched cellular components. **B** Ten most significantly enriched pathways. Gene ratio is the ratio of the number of genes in the query list and the hit count for that gene set in the genome

As the majority of DMPs had effect sizes below 5%, we conducted gene set enrichment analysis (GSEA) using *ToppGene* on all DMPs that mapped to a gene (812 of 965, 84%) to elucidate potentially cumulative effects of many DMPs with small effect sizes. *ToppGene* revealed that differential methylation, regardless of direction of effect, was primarily enriched in the three cellular components: synapse (*n*_genes_ = 101, FDR_B&H_ = 1.53 × 10^–5^), supramolecular complex (n_genes_ = 116, FDR_B&H_ = 1.53 × 10^–5^) and glutamatergic synapse (*n*_genes_ = 46, FDR_B&H_ = 1.53 × 10^–5^, Fig. 3A, Additional file 2: Table S12). The top enriched pathway was B cell receptor signalling (*n*_genes_ = 15, FDR_B&H_ = 0.00098, Fig. 3B**,** Additional file 2: Table S12), and notable pathways included the p75 neurotrophin receptor (NTR)-mediated signalling (*n*_genes_ = 13, FDR_B&H_ = 0.016**,** Additional file 2: Table S12) and NRAGE signals death through c-Jun N-terminal kinases (JNKs) (*n*_genes_ = 11, FDR_B&H_ = 0.007**,** Additional file 2: Table S12).

**Fig. 4:**
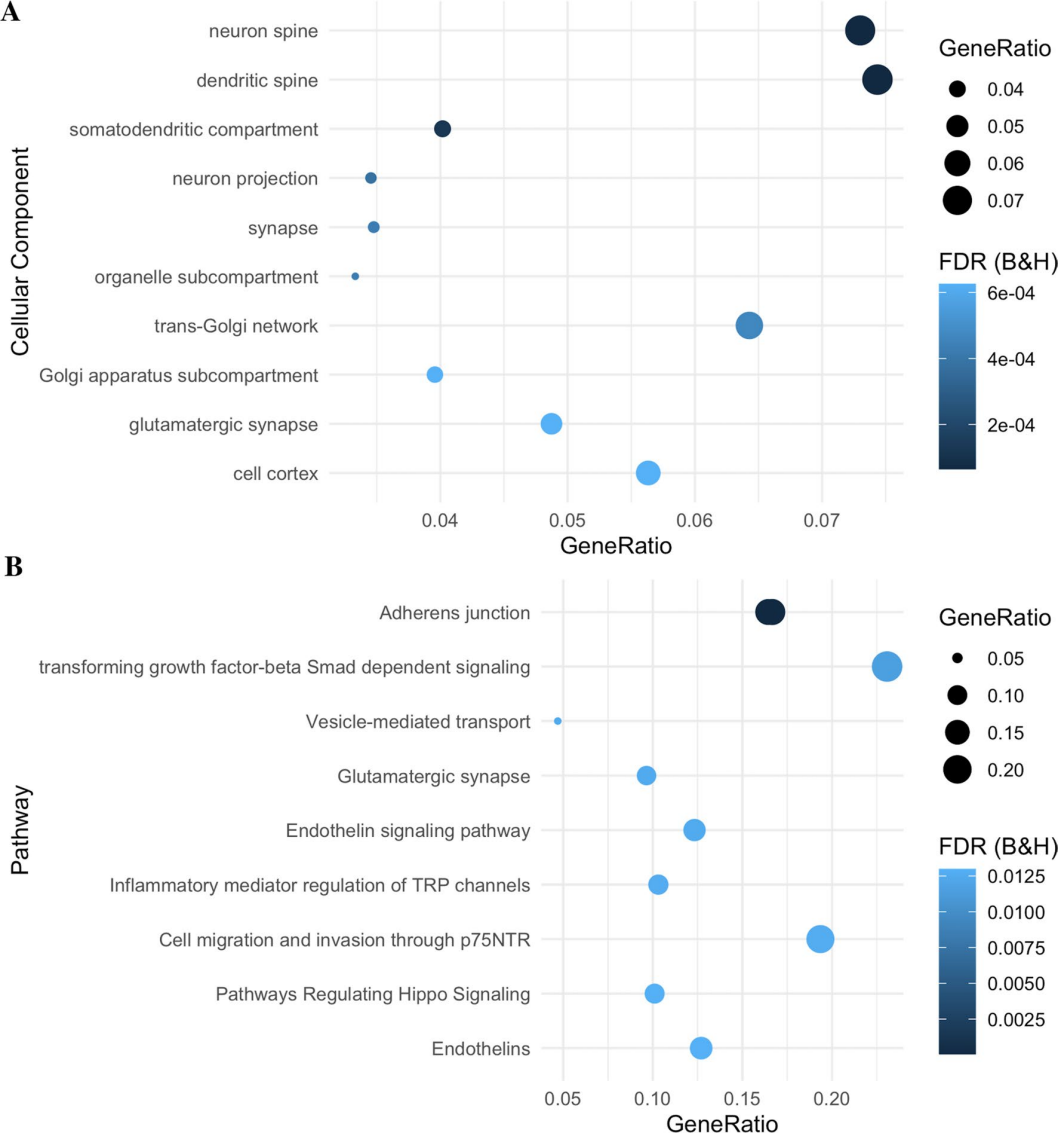
Gene Set Enrichment Analysis (GSEA) of hypermethylated differentially methylated positions (DMPs). GSEA was conducted using 391 hypermethylated genes. **A** Ten most significantly enriched cellular components. **B** Ten most significantly enriched pathways. Gene ratio is the ratio of the number of genes in the query list and the hit count for that gene set in the genome

**Fig. 5:**
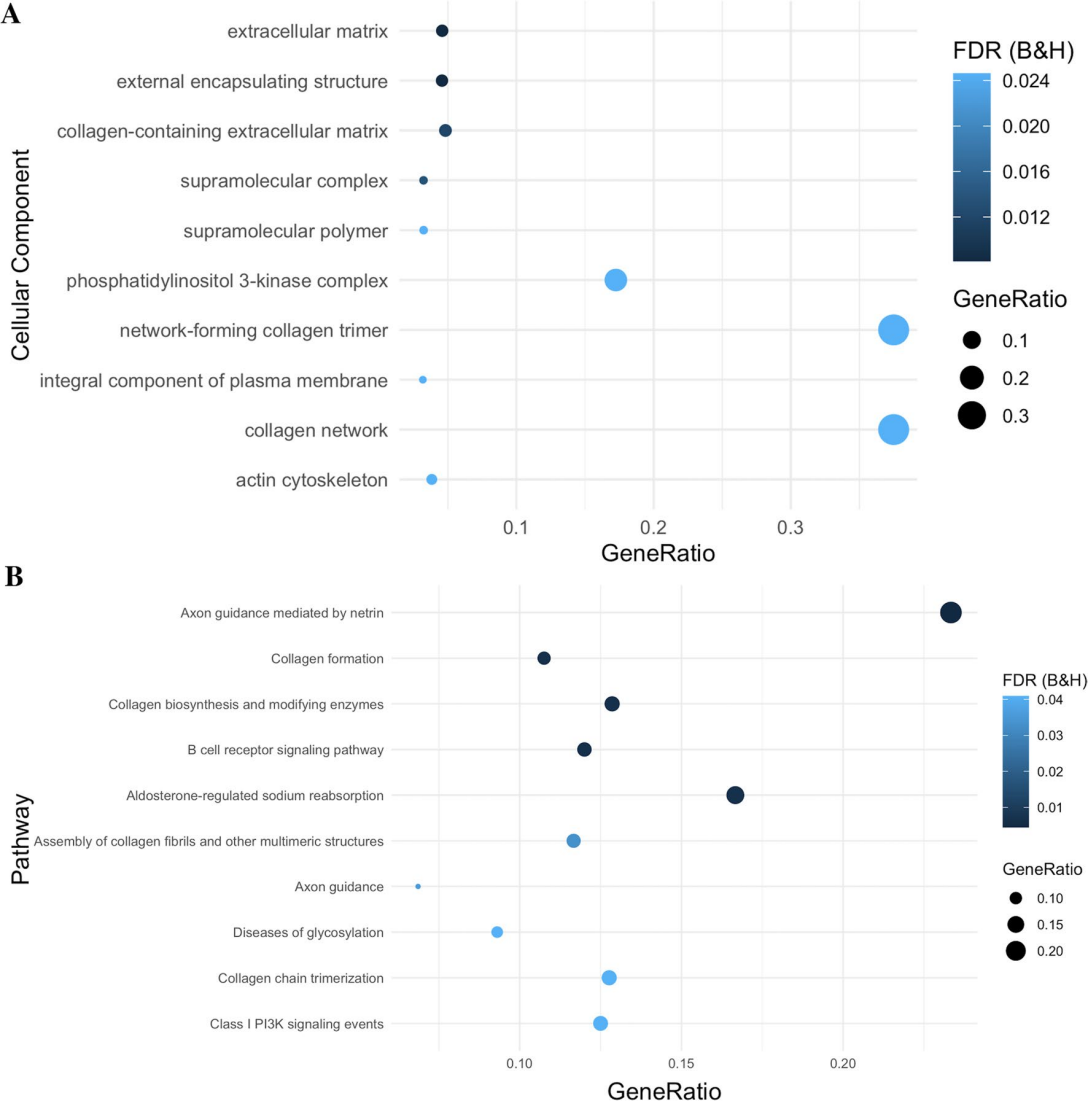
Gene Set Enrichment Analysis (GSEA) of hypomethylated differentially methylated positions (DMPs). GSEA was conducted using 440 hypermethylated genes. **A** Ten most significantly enriched cellular components. **B)** Ten most significantly enriched pathways. Gene ratio is the ratio of the number of genes in the query list and the hit count for that gene set in the genome

Hypermethylated genes (*n* = 391) were enriched in neuron related cellular components such as neuron spine (*n*_genes_ = 20, FDR_B&H_ = 6.48 × 10^–5^) and dendritic spine (*n*_genes_ = 20, FDR_B&H_ = 6.48 × 10^–5^, Fig. 4A**,** Additional file 2: Table S13), as well as the Adherens junction pathway (*n*_genes_ = 12, FDR_B&H_ = 4.81 × 10^05^, Fig. 4B**,** Additional file 2: Table S13). Hypomethylated genes (*n* = 440) were enriched in extracellular matrix (*n*_genes_ = 29, FDR_B&H_ = 0.00813), and external encapsulating structure cellular components (*n*_genes_ = 29, FDR_B&H_ = 0.00813, Fig. 5A**,** Additional file 2: Table S14), and the axon guidance mediated by netrin (NTN) pathway (n_genes_ = 9, FDR_B&H_ = 0.0006) and B cell receptor signalling pathway (*n*_genes_ = 7, FDR_B&H_ = 0.00443, Fig. 5B**,** Additional file 2: **Table S14**).

### Disease severity is associated with methylation age acceleration using PhenoAge

Methylation age is the estimation of biological age from methylation levels at a subset of CpGs associated with age, known as clock CpGs. Using two validated methylation age algorithms (PhenoAge [22] and GrimAge [23]) we did not find any evidence for significant differences in methylation age between severity groups (PhenoAge, *p* = 0.064; GrimAge, *p* = 0.343; data not shown). We did identify two PhenoAge clock CpGs as DMPs in our study (Additional file 2: Table S15). However, this overlap did not meaningfully contribute to differences in MAA between severity groups.

Methylation age acceleration (MAA) is a measure of the disparity between chronological and biological age, and can provide insight into an individual’s state of health and potential lifespan [22–24]. It is defined as the residual term from regressing chronological age on methylation age. The distribution of residual terms was normal for PhenoAge (*p* = 0.599) and GrimAge (*p* = 0.508), so t-tests were used to assess mean difference in MAA between mild and severe groups. There were significant differences in MAA between mild and severe groups using PhenoAge (Δμ = 1.36, *p* = 0.048), but not GrimAge (Δμ = 0.464, *p* = 0.375; Additional file 2**Fig. S8**).

Sex, smoking history, body mass index (BMI) and socioeconomic status (SES) are reported confounders of PhenoAge[25]. To investigate whether the disparate findings between PhenoAge MAA and GrimAge MAA were due to unaddressed confounder effects, we compared smoking history between sample groups. Mild and severe groups showed no significant differences in DNA methylation smoking pack years (DNAmPACKYRS, *p* = 0.4342, data not shown). Sex did not need to be addressed as all participants are female, while BMI and SES data was not available for this cohort.

### Whole-blood methylation levels classify disease severity more accurately than clinical data

To understand the potential of methylation as a predictive biomarker of severity with clinical utility, we compared the accuracy of three models in classifying binary disease severity in our cohort using elastic net regression. Elastic net regression is a form of penalised regression that is useful for datasets with correlated features (e.g. methylation) and a greater number of features than subjects. Model 1 used clinical data available at diagnosis, including age at onset (AAO) and first symptoms (optic pathways, supratentorial, brainstem and/or spinal cord). Model 2 used genome-wide methylation data at 747,969 CpGs and immune cell-type proportion estimates. Model 3 used a weighted methylation risk score (wMRS) of DMPs identified in the main differential methylation analysis (*n* = 1472). All models were trained on 70% of the cohort (*n* = 164).

**Fig. 6:**
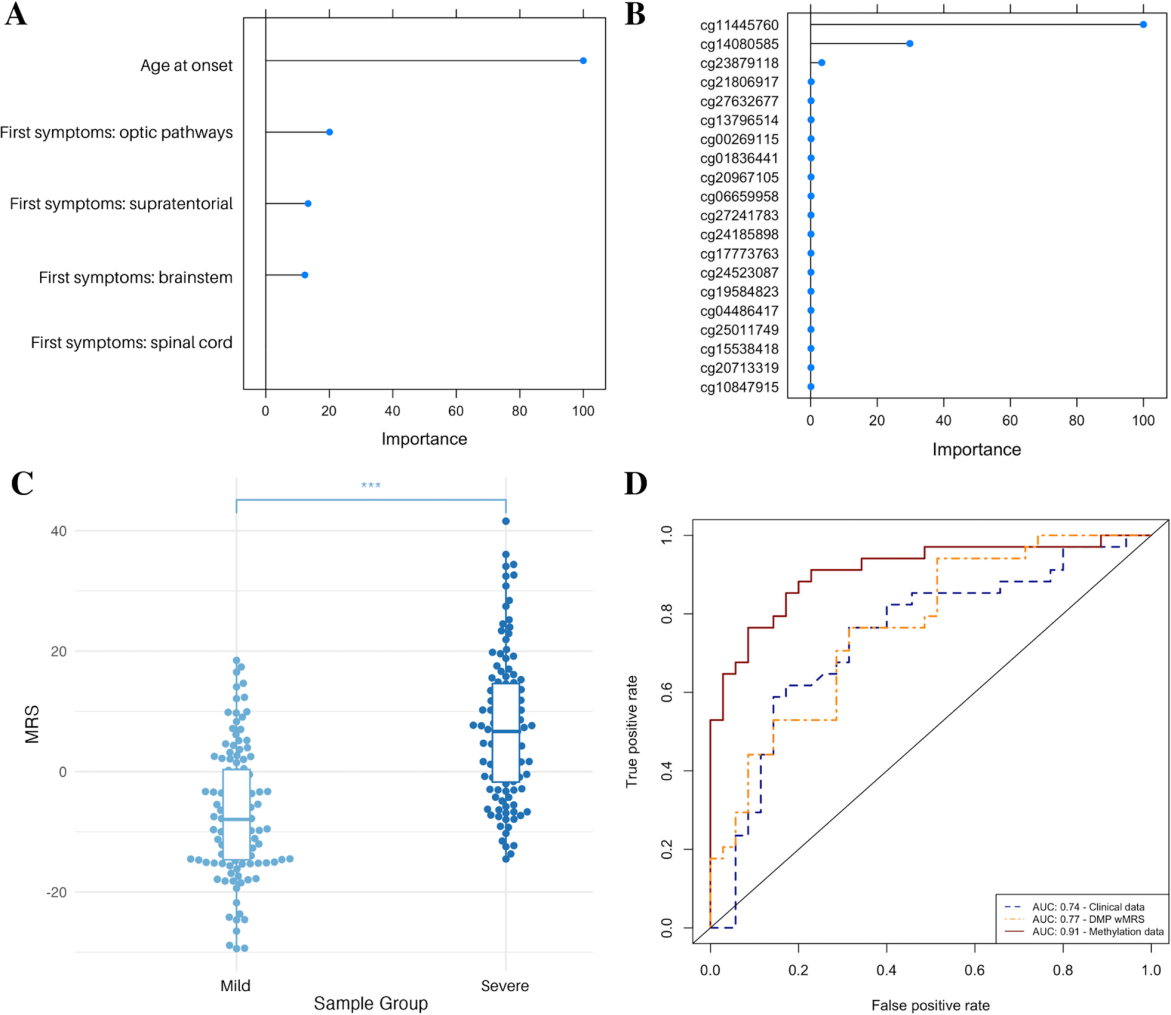
Multifactor feature selection and classification modelling using clinical or methylation data. **A** Variable importance plot for model 1 (clinical data available at diagnosis). **B** Variable importance plot for model 2 (methylation data at 1708 CpGs). **C** Distribution of weighted methylation risk scores (wMRS) by Sample Group (*p* < 2.72 × 10^–16^). **D** Receiver Operator Curves (ROCs) of disease severity classification using clinical data (AUC = 0.74), methylation data at 1708 CpGs (AUC = 0.91) or wGRS (AUC = 0.77). Models were trained on 164 samples (70%) and tested on 71 samples (30%)

For model 1 and 2, we used a cross-validation approach to determine the optimal alpha and lambda values (model 3: alpha = 0.4, lambda = 0.20, model 3: alpha = 0.01, lambda = 0.02). Using these parameters model 1 selected all five clinical variables (Fig. 6A), with AAO as the most important clinical factor for classifying disease severity. Model 2 selected 1708 CpGs associated with disease severity (Fig. 6B, top 10 shown in Table 4, full list in Additional file 2: Table S16). The most important CpG in model 3, cg11445760 (variable importance = 100), is located on Chromosome 11 and maps to the Splicing Factor 1 (SF1) gene. cg11445760 is not located in the DMR we identified on the same chromosome (DMR^Chr11^) in the primary differential methylation analysis. The 1708 CpGs in model 2 mapped to 1018 genes strongly enriched in nerve growth factor (NGF) signalling pathways (*n*_genes_ = 56, FDR_B&H_ = 2.68 × 10^–5^, Additional file 2: Table S17). Comparison of features in model 2 with DMPs identified in the main differential methylation analysis highlighted five overlapping CpGs and 100 overlapping genes (Additional file 2: Table S18). Overlapping genes were enriched in dendrite extension biological processes (*n*_genes_ = 5, FDR_B&H_ = 4.94 × 10^–3^) and neuron projection cellular components (*n*_genes_ = 21, FDR_B&H_ = 9.736 × 10^–3^, Additional file 2: Table S19). In model 3, the wMRS was significantly different between mild and severe groups (*p* < 2.73 × 10^–17^, Fig. 6C).

**Table 4:** Top ten CpGs associated with disease severity as selected by the elastic net model

CpG	Importance	Chr	Position (bp)	Gene
cg11445760	100.000	11	64546351	*SF1*
cg14080585	29.792	20	60639721	*TAF4*
cg23879118	3.324	11	67414373	*ACY3*
cg21806917	0.137	11	66384012	*RBM14*
cg27632677	0.106	2	114385169	*RPL23AP7*
cg13796514	0.106	19	39881827	*PAF1*
cg00269115	0.102	1	23695530	*C1orf213*
cg01836441	0.102	12	70636618	*CNOT2*
cg20967105	0.096	2	64069295	*UGP2*
cg06659958	0.089	7	149126002	

*Abbreviations: Chr* = *Chromosome, bp* = *base pairs*

We tested the classification capacity of each model on the remining 30% of the cohort (*n* = 71). Model 2 classified disease severity the most accurately (AUC = 0.91), followed by model 2 (AUC = 0.77) and model 1 (AUC = 0.74, Fig. 6D).

## Discussion

There is limited, but growing evidence of association between genetics and disease severity in MS [5], but well established relationships between environmental variables such as smoking [7], DMT exposure [9] and pregnancy [9] on long-term outcomes. Despite this, no published studies to date have examined the association between disease severity and epigenetic mechanisms, such as DNA methylation. We hypothesised that MS severity is associated with DNA methylation. To test this, we compared genome-wide methylation between 119 relapse-onset females with mild disease and 116 age-matched females with severe disease. Disease severity was determined with median longitudinal ARMSS scores using data from the MSBase Registry. We identified numerous differentially methylated CpG sites across the genome with small, but likely cumulative impacts on MS severity. We also identified differential methylation in immune cell types, mainly CD8+ T cells, using reference-based statistical deconvolution. Gene set enrichment analyses identified neuronal, rather than immune pathways, as differentially methylated between outcome extremes. Finally, using a machine learning approach, we were able to accurately assign disease severity demonstrating the potential of methylation as a prognostic biomarker in MS.

Our analysis of whole-blood methylation differences between groups at outcome extremes identified 1472 DMPs, the majority of which had small effect sizes below 5% (*n* = 1455). Of the 1472 DMPs, 55% mapped to genes enriched in CNS cellular components and pathways. Notably, hypermethylated genes were enriched in neuronal structures, including neuron spine, dendritic spine and neuronal projection. Recent studies show the specific vulnerability of excitatory projection neurons to chronic cortical inflammation, compared to inhibitory neurons [26]. Our findings lend support to a recent suggestion that excitatory cortical projection neurons could be a novel therapeutic target to combat MS-related neurodegeneration [27]. Hypomethylated genes in the severe group were enriched in axon guidance pathways. Research in both humans and mouse models of MS have shown that levels of NTN1, an axon guidance gene that reduces immune cell translocation into the CNS, are lower in MS compared to healthy comparators, particularly during relapse [28]. Further to this, the inhibition of repulsive guidance molecule-a, an axon guidance gene that prevents axon growth and immune regulation, has shown therapeutic efficacy in MS animal models [29]. In our study, we have shown hypomethylation, and subsequently potentially increased expression, of axon guidance genes in patients with severe disease. Although counterintuitive, hypomethylation of axon guidance pathways in those with severe MS may represent an adaptive mechanism aimed at countering MS-associated neurodegeneration, where neurodegenerative pathways regulated via the p75 neurotrophin receptor, and NRAGE-mediated cell death pathways were also identified as differentially methylated in our study. These findings add to a growing body of evidence implicating neuronal response to injury in disease severity. Jokubaitis et al. recently identified genetic variants associated with disease severity based on longitudinal ARMSS scores overrepresented in synaptic plasticity processes [6], while Kosa et al. identified synaptogenesis as a pathway differentially activated between patients with different levels of disability based on spinal cord damage [30].

We further showed that hypomethylated genes were enriched in the B cell receptor signalling pathway. Hypomethylation may indicate increased expression of B cell receptor signalling pathway genes, leading to more severe disease. There is a strong basis for a role of B cells in MS pathogenesis [31], further supported by the efficacy of monoclonal anti-CD20 antibodies as DMTs, by selectively depleting CD20+ B cells primarily via antibody-dependent cell-mediated cytotoxicity [32]. Given the high rates of response to B cell depleting therapies in patients with severe MS [32], it is unsurprising that this signalling pathway is implicated somewhat in disease severity. The enrichment of the B cell receptor signalling pathway in this study, paired with the efficacy of B cell depleting DMTs implicate a role for B cells in disease severity, additional to their putative role in pathogenesis [32]. Overall, our results support a dichotomy between mechanisms regulating MS risk and MS outcome, where we have overarchingly identified neurodegeneration-related pathways, rather than immune-related pathways, as dysregulated in severe MS. This is consistent with the current understanding of progressive MS, as demonstrated by the lack of effectiveness of most immunosuppressive treatments for secondary and primary progressive MS.

We identified two differentially methylated regions (DMRs) in whole-blood. DMR^Chr15^ (Chr15:57,664,490–57,664,704) was hypermethylated in the severe group and located in an intergenic region. DMR^Chr11^ (Chr11:94,886,261–94,886,708) contained two DMPs that were hypomethylated in the severe group. While cg10357314 is in an intergenic region, cg02877698 is in the gene body of uncharacterised long noncoding RNA (lncRNA) gene *LOC101929295*. lncRNAs are genes that are transcribed, but never translated (noncoding), as their primary role is in transcriptional, post-transcriptional and translational regulation of gene expression [33]. Numerous studies have described a role for lncRNAs in MS pathogenesis through the alteration of immune cell differentiation and activation [34]. Gupta et al. (2019) demonstrated the prognostic potential of lncRNAs by identifying eight upregulated lncRNAs in severe (*n* = 21) RRMS cases relative to mild (*n* = 43) RRMS cases. They also categorised disease severity using longitudinal ARMSS scores. Gupta et al. did not identify *LOC101929295* as an upregulated lncRNA. It is possible that we did not validate the findings of Gupta et al. as our cohort was sex-matched and much larger, or due to post-transcriptional modifications. Nevertheless, when taken together these results demonstrate a potential role for epigenetically regulated lncRNA in disease severity.

Targeted mQTL analysis of major DMPs and DMRs identified genetic effects on methylation at these loci. However, these genetic effects did not attenuate the association between methylation and disease severity. It must be noted that the genetic architecture of the methylome is highly complex with many CpGs being influenced by both cis and trans acting mQTLs [35]. For this reason, we cannot rule out more complex genetic interactions with the major DMPs and DMRs identified in this study.

We identified cell-specific methylation differences between mild and severe patients by estimating cell-type proportions from whole-blood methylation data using statistical deconvolution. The majority of csDMPs were in CD8+ T cells (*n* = 48), a handful of which mapped to genes shown to be differentially expressed in animal models of neurodegenerative diseases (e.g. Dynactin Subunit 5[36], Isopentenyl-Diphosphate Delta Isomerase 1 [37], SLIT-ROBO Rho GTPase Activating Protein 1 [38] and Phosphodiesterase 4D [39]). Given the absence of DMRs in CD8+ T cells, and low number of csDMPs in other immune cell types, these results serve to guide future cell-type-targeted studies of disease severity, rather than demonstrating a mechanistic role for individual cell-type methylation in determining clinical outcomes.

Methylation age acceleration (MAA) is associated with increased all-cause morbidity and mortality. We identified slower MAA in mild patients, compared to severe, using the PhenoAge algorithm. We did not observe the same effect with the GrimAge algorithm which has been shown to be more robustly associated with clinical outcomes in comparison to PhenoAge, including walking speed, polypharmacy, frailty and mortality [25]. However, different results between the algorithms are not unexpected as they were designed using different clinical markers as ageing measures [25]. PhenoAge uses albumin, creatinine, serum glucose, c-reactive protein, lymphocyte per cent, mean cell volume, red cell distribution width, alkaline phosphatase and white blood cell count [22], while GrimAge uses adrenomedullin, beta-2-microglobulin, cystatin C, growth differentiation factor 15, leptin, plasminogen activation inhibitor 1, tissue inhibitor metalloproteinase 1 and smoking pack years [23]. It is possible that methylation patterns between mild and severe patients differ more at DNA methylation proxies of PhenoAge clinical variables, compared to the GrimAge clinical variables. Notably, the PhenoAge clinical variables lymphocyte per cent and white blood cell count are impacted by DMTs for MS. Therefore, PhenoAge may be more sensitive to methylation differences in people with MS than GrimAge. Alternatively, the disparity between PhenoAge and GrimAge acceleration in this paper may be driven by unaccounted confounder effects, including smoking history, BMI and SES [25]. We tested smoking history using an estimate of DNA methylation smoking pack years and showed no significant differences in smoking pack years between mild and severe groups. While differences in smoking history are not driving the significant differences in PhenoAge MAA between groups, we could not account for confounding by BMI and SES due to lack of data.

To understand the potential of whole-blood methylation as a prognostic biomarker, we compared the accuracy of three models in classifying binary disease severity. One model used only clinical data available at diagnosis (model 1), while two models used methylation data in the form of selected features from elastic net regression (model 2) or a weighted methylation risk score (wMRS) of DMPs (model 3). The importance of age at onset (AAO) in model 1 confirms current understanding that later AAO is associated with greater long-term disability [9]. For model 2, elastic net regression identified whole-blood methylation levels at 1708 CpGs to be associated with disease severity, of which five overlapped with the main differential methylation analysis at the CpG level and 100 CpGs overlapped at the gene level. These genes were enriched in neuronal components and processes, adding to a growing evidence that disease severity is driven by neuronal, rather than immune, mechanisms [6, 30]. The ability of the machine learning-trained elastic net regression model to identify 1702 additional DMPs to the main analysis demonstrate the presence of small, correlated and potentially cumulative effects of methylation on disease severity. While this requires a larger cohort to understand at a more granular level, it emphasises the value of accounting for correlated effects across the genome using methods such as penalised regression.

Model 2 classified disease severity the most accurately (AUC = 0.91). The improved accuracy of classifying disease severity using methylation levels at correlated CpGs (model 2), compared clinical data available at diagnosis (model 1), demonstrates the potential of whole-blood methylation as a clinical biomarker. At diagnosis, clinicians prognosticate based on AAO, presenting symptoms and MRI lesions. Our findings lend support to a growing body of evidence that genomic signatures may help clinicians prognosticate more accurately than clinical data alone [6]. We are confident that our models are not overfitted due to internal validation using training and testing sets with a conservative 70:30 split. Nevertheless, validation of the model in an independent mixed-sex cohort at diagnosis would confirm the robustness and generalisability of our multi-SNV signature as a true prognostic biomarker.

Ours is the largest study to date examining severity-associated differential methylation in a cohort of females with RMS. Further, ours is the only study reported to date that has utilised longitudinal severity data to ascribe severity status. Given the fluctuation of EDSS scores across disease course [40], the ability to robustly phenotype patients using longitudinal data, rather than cross-sectional data, reduces the probability of erroneous associations. Categorising disease severity by ARMSS score also allows comparison of cohorts a priori adjusted for age, a known factor that may confound methylation analyses. Our findings require validation in an independent cohort of females and, further, extension to males to confirm that differential methylation patterns between those with mild and severe RMS is consistent between sexes. The prognostic utility of correlated methylation patterns requires independent validation, although we did try to mitigate against overfitting using a 70:30 training:testing strategy. A range of environmental factors impact methylation, and we were underpowered to adjust for many of these, including BMI, SES and DMT at blood collection [41].

## Conclusion

This is the first study to investigate the association between genome-wide methylation patterns and longitudinal disease severity in relapse-onset MS. We identified differences in whole-blood methylation patterns between 119 mild and 116 severe females with RMS, and demonstrate a dichotomy in signally pathways associated with MS severity (enriched in neuronal pathways and signalling) as opposed to past studies of risk. Using a machine learning strategy, we show that correlated methylation patterns classify disease severity more accurately than clinical data. Our findings support evidence that genomic signatures may help clinicians prognosticate more accurately than clinical data alone at diagnosis. Validation studies are needed to confirm our findings and assess the utility of differential methylation as a prognostic biomarker in relapse-onset MS.

## Materials and methods

### Clinical data collection

This study utilised clinical data from the MSBase Registry, an international, prospective, observational MS clinical outcomes register. Data are collected in a unified manner and include patient demographics, EDSS scores, relapse and treatment data, as previously described [42].

### Participant recruitment, severity definitions and sample collection

Using MSBase Registry data, we assessed patients for study eligibility based on the following criteria: a diagnosis of relapse-onset MS, European ethnicity, female, Australian, minimum five years of clinical follow-up, minimum three relapse-independent EDSS scores recorded, and available genotype and whole-blood methylation data. We restricted participants to Australian females to reduce confounding by sex and geographical location as these are known to impact methylation [43].

Disease severity was determined using a longitudinal ARMSS [18] approach, as previously described [6]. Briefly, ARMSS scores were calculated for each relapse-independent Expanded Disability Status Scale (EDSS) score [19] available, and the median of these longitudinal ARMSS scores was calculated. Patients were categorised as mild or severe based on median longitudinal ARMSS scores. Mild patients were those with median longitudinal ARMSS scores at or below the cohort 20th percentile, while severe patients were those at or above the cohort 80th percentile. Our final cohort consisted of 119 mild patients and 116 age-matched severe patients (*n* = 235, Additional file 1: Fig. S1). Included study sites were Royal Melbourne Hospital (VIC, *n* = 81), Box Hill Hospital (VIC, *n* = 67), Flinders Medical Centre (SA, *n* = 51) and John Hunter Hospital (NSW, *n* = 36).

### Methylation arrays

Whole-blood genomic (gDNA) was processed for methylation arrays at the Hunter Medical Research Institute. The Qbit (Invitrogen™, USA) and TapeStation (Agilent™, USA) were used to assess DNA quantity and quality, respectively. Samples were bisulfite converted using the EZ-DNA Methylation™ Kit (Zymo) kits and hybridised to Illumina MethylationEPIC BeadChip arrays (EPIC arrays). To avoid batch effects, samples were randomised by clinic site using the *OSAT* R package. We used an iScan (Illumina™) to read the EPIC arrays, which produced raw Idat files for analysis.

### Genotyping arrays

Genotyping was performed at the Center for Genome Technology, John P. Hussman Institute for Human Genomics, University of Miami using Illumina Multiethnic genotyping arrays (MEGA^EX^). Genotype calling was performed using GenomeStudio v2.0 (Illumina).

### DNA methylation analysis pipeline

We used the *ChAMP* Bioconductor package (version 2.22.0) [20] for methylation data preprocessing in the R statistical environment. We filtered raw Idat files to exclude low-quality samples (failed to successful probe ratio > 0.1), low-quality probes (detection *p* value > 0.01, bead count < 3 in ≥ 5% of samples), non-CpG probes, SNP-related probes, non-autosomal probes and multi-hit probes. We additionally excluded multi-hit probes based on Pidsley (2016) Additional file 2:Table S1 [44]. We normalised methylation beta values using the Beta-Mixture Quantile (BMIQ) method [45]. We identified batch effects at the array and chip level using singular value decomposition (SVD) analysis [46] and corrected these effects using the *Combat* algorithm [47].

### Primary differential methylation analysis

We identified differential methylation between mild and severe groups at the single CpG level (i.e. differentially methylated positions (DMPs)) and at the genomic region level (i.e. DMRs). We implemented a linear model of methylation level at each probe and severity group, adjusted for NK cell proportions, using a filtered and normalised beta matrix as input. This was a modified version of the function *champ.DMP* from the *ChAMP* R package. We used a false discovery rate (FDR) threshold of 0.05 to assess statistical significance for all analyses. Methylation beta values equate to methylation percentage (i.e. Δ_meth_ of 0.01 = 1%), therefore, we report methylation differences (Δ_meth_) in percentages below. To avoid false positives driven by technical error, we removed DMPs with an Δ_meth_ < 1%.

We used two methods to identify DMRs. Firstly, the *DMRcate* R package (version 2.2.3) [48] with the following parameters: at least three DMPs within 1000 bp of the adjacent DMP, a DMP threshold of FDR < 0.05, an DMR threshold of FDR < 0.05. Secondly, DMRs were identified from the DMP list as ≥ 2 DMPs with (1) an FDR < 0.01, (2) the same direction of effect and (3) located within 1000 bp. Previous studies demonstrate the robustness of this strategy to identify DMRs in studies with small sample and/or effect sizes [12, 16].

Methylation patterns can be cell-type-specific. Therefore, we estimated immune cell-type proportions to confirm that differential methylation in whole-blood was not driven by cell-type proportion differences between groups. We estimated immune cell-type proportions with the *EpiDISH* R package (version 2.8.0) [49] and reference-based *CIBERSORT* algorithm [21], using methylation M-values as input. We then used a modified version the *cellDMC* function of *EpiDISH*, to identify csDMPs. Here, the outcome term of a linear model was methylation M-value, and the predictors were cell-type proportion estimate and an interaction term of cell-type proportion and severity. A genome-wide threshold of *p* ≤ 9 × 10^–8^ was used to assess statistical significance.

### Sensitivity analyses

Methylation patterns are impacted by a range of clinical and environmental factors. Therefore, we performed sensitivity analyses to assess the potential impact of a series of demographic, clinical, biological and environmental covariates on methylation patterns, including: age at blood collection, symptom duration, annualised relapse rate, cell-type proportion estimates (B cells, CD4+ cells, CD8+ cells, NK cells, neutrophils and eosinophils) and methylation age acceleration. Additional categorical environmental factors were assessed, including treatment at blood collection (yes or no, specific treatment at blood collection available in Additional file 2: Table S20), smoking status at blood collection (ever or never) and parity at blood collection (nulliparous or parous). An FDR threshold of 0.05 was used to assess statistical significance.

Mild and severe patients were matched by age at blood collection (*n* = 214, 107 pairs), and the difference in methylation at each probe (Δ_meth_) was calculated. The correlation between each Δ_meth_ and covariate was tested. Pearson’s correlation was used for continuous covariates and ANOVA for categorical covariates. For categorical variables (treatment, smoking history, parity), mild–severe pairs were required to have the same value for the correlation with methylation to be tested. Of 107 pairs in total, 17 pairs were on treatment at blood collection and 12 were off treatment, 25 pairs were nulliparous at blood collection and 17 were parous, and six pairs were ‘ever’ smokers at blood collection and four were ‘never’ smokers (Additional file 2: Table S21). Due to the known effect of smoking on the methylome and limited smoking data available for this cohort, DMPs were filtered for 2,622 known smoking-associated CpGs identified by Johanes and colleagues (2016) [50].

### Single-nucleotide variant analysis

We performed quality control with *PLINKv1.9 *[51]. Single-nucleotide variants (SNVs) were excluded based on low call rate (< 95%), low minor allele frequency (MAF < 0.05), violation of Hardy–Weinberg equilibrium (*p* < 1 × 10^–5^), monomorphism and non-autosomal location. Samples were excluded based on sex inconsistencies, low call rate (< 95%) and relatedness (pi-hat > 0.05). We assessed relatedness using Identity by Descent (IBD) analysis in *PLINKv1.9*, followed by confirmation in *KING* [52]. Principal components (PC) analysis was implemented in *EIGENSTRAT *[53]. We projected PCs to 1000 Genomes Project [54] data to assess population stratification effects and exclude population outliers. We imputed genotypes using the Haplotype Reference Consortium [55] on the Michigan Imputation Server (https://imputationserver.sph.umich.edu/index.html#!) and converted imputed genotypes to genotype calls in *PLINKv1.9*.

### Targeted methylation quantitative trait loci (mQTL) analysis

To account for genetic effects on methylation levels, we conducted a targeted mQTL analysis by testing the relationship between genotype and methylation levels within a) a ± 5 kb window of each major DMP (i.e. Δ_meth_ > 5%) and b) each DMR.

We used the *KRIS* R package (version 1.1.6)[56] to extract SNVs located within a ± 5 kb window of each major DMP and within the boundaries of each DMR. For SNVs on the same chromosome, we assessed linkage disequilibrium (LD) using bivariate correlations of genotype frequencies using a statistical significance threshold of p < 0.05.

We then assessed the relationship between methylation level (beta values) and genotype using Kruskal–Wallis tests due to the beta binomial (non-normal) distribution of methylation beta values. Lastly, we used binary logistic regression with severity group as the outcome and methylation and genotype as the predictors, to test whether differential methylation at major DMPs/DMRs were regulated by genetic effects rather than disease severity.

### Gene set enrichment analysis (GSEA)

We performed gene set enrichment analysis (GSEA) with the the *ToppGene* online application programming interface (API) to explore biological processes and pathways enriched by differentially methylated genes. We used an FDR-ranked genes as input and analysed hypomethylated and hypermethylated genes both together and separately. A Benjamini–Hochberg adjusted *p* value threshold ≤ 0.05 was used to assess the statistical significance.

### Methylation age acceleration analysis

Methylation age is the estimation of biological age from methylation levels at a subset of CpGs associated with age, known as clock CpGs. Multiple algorithms have been developed to perform this estimation, the most widely used algorithms being PhenoAge [22] and GrimAge [23]. Methylation age acceleration (MAA) is defined as the discrepancy between chronological and biological age, whereby an acceleration of biological age is associated with increased risk of various morbidities and mortality, as well as shorter lifespan [22–24].

We estimated the PhenoAge [22] of our samples with the *methyAge* function of the *ENmix* R package (version 2.8.0) [57]. GrimAge was calculated with the online calculator at https://dnamage.genetics.ucla.edu/. MAA was defined as the residual term from regressing chronological age on methylation age estimates. We used Shapiro–Wilk normality tests to assess the normality of each MAA distribution and t-tests to assess mean difference in MAA between mild and severe groups.

To test smoking history as a potential confounder of PhenoAge, we used DNA methylation smoking pack years (DNAmPACKYRS) as a measure of smoking history, calculated using the GrimAge online tool: https://dnamage.genetics.ucla.edu/home. Shapiro–Wilk normality tests were used to assess the normality of the DNAmPACKYRS distribution in each group, and a Wilcoxon signed rank test was used to assess mean difference in DNAmPACKYRS between mild and severe groups.

### Multifactor feature selection and classification modelling

For each model, we split samples into training (*n* = 164) and testing sets (*n* = 71) to limit overfitting. For models 1 and 2, we conducted multifactor feature selection in the training set using a cross-validation resampling method with 10 iterations via the train function of the *caret* R package (version 6.0–93) [58]. We then conducted a k-fold cross-validation elastic net regression using the optimal alpha value to identify the minimum lambda value with the *cv.glmnet* function of the *glmnet* R package (version 4.1–4) [59]. These alpha and lambda values were used in the final elastic net regression models that were applied to the testing set using the *glmnet* function [59]. We compared the CpGs selected in model 2 with DMPs from the main differential methylation analysis at the CpG and gene level. GSEA of the overlapping genes were performed using *ToppGene*.

For model 3, we calculated a weighted methylation risk score (wMRS) for each sample:

$$ {\text{wMRS}}_{{{\text{sample}}}} = \sum \beta z - {\text{score}}_{{{\text{Sample}} {\text{at}} {\text{DMPi}}}} \times \Delta \beta_{{{\text{DMPi}}}} $$

The wMRS for each sample was subsequently used in a general linear model as the predictor, with sample group as the outcome.

We tested the ability of each model to classify test samples as mild or severe using base R function, *predict*(). The *performance* function of the *ROCR* R package (version 1.0–11) [60] was used to test the performance of the classification model with the AUC measure.

## Supplementary Information

**Additional file 1.**. Supplementary figures.

**Additional file 2.**. Supplementary tables.

## Data Availability

Supplemental files contain data supporting the conclusions in this article. For access to deidentified raw methylation data and code please contact Dr Vilija Jokubaitis (vilija.jokubaitis@monash.edu). Data sharing requests will be reviewed based on a research proposal. Sharing de-identified patient-level clinical data from MSBase Registry may be possible in principle, but will require permission/consent from each contributing data controller to protect participant confidentiality.

## Abbreviations

ARMSS: Age-related multiple sclerosis severity
ChAMP: Chip analysis methylation pipeline
CpG: Cytosine–phosphate–guanine
csDMP: Cell-specific differentially methylated position
DMP: Differentially methylated position
DMR: Differentially methylated region
DMT: Disease-modifying therapy
EDSS: Expanded disability status scale
EWAS: Epigenome-wide association study
FDR: False discovery rate
GSEA: Gene set enrichment analysis
GWAS: Genome-wide association study
JNK: c-Jun *N*-terminal kinase
lncRNA: Long noncoding RNA
MAA: Methylation age acceleration
mQTL: Methylation quantitative trait loci
MS: Multiple sclerosis
NGF: Nerve growth factor
NK: Natural Killer
NTN: Netrin
NTR: Neurotrophin receptor
PPMS: Primary progressive multiple sclerosis
RMS: Relapse-onset multiple sclerosis
RRMS: Relapsing–remitting multiple sclerosis
wMRS: Weighted methylation risk score
SF1: Splicing factor 1
SNP: Single-nucleotide polymorphism
SPMS: Secondary progressive multiple sclerosis
