# Supplementary material for: Whole-blood methylation signatures are associated with and accurately classify multiple sclerosis disease severity
Source: Clin Epigenetics. 2022 Dec 30;14:194. doi: 10.1186/s13148-022-01397-2 (PMC9805090; doi:10.1186/s13148-022-01397-2)
Supplement: Supplementary file 1 — Additional file 1.. Supplementary figures. [file 13148_2022_1397_MOESM1_ESM.docx]

**Supplementary Figures**


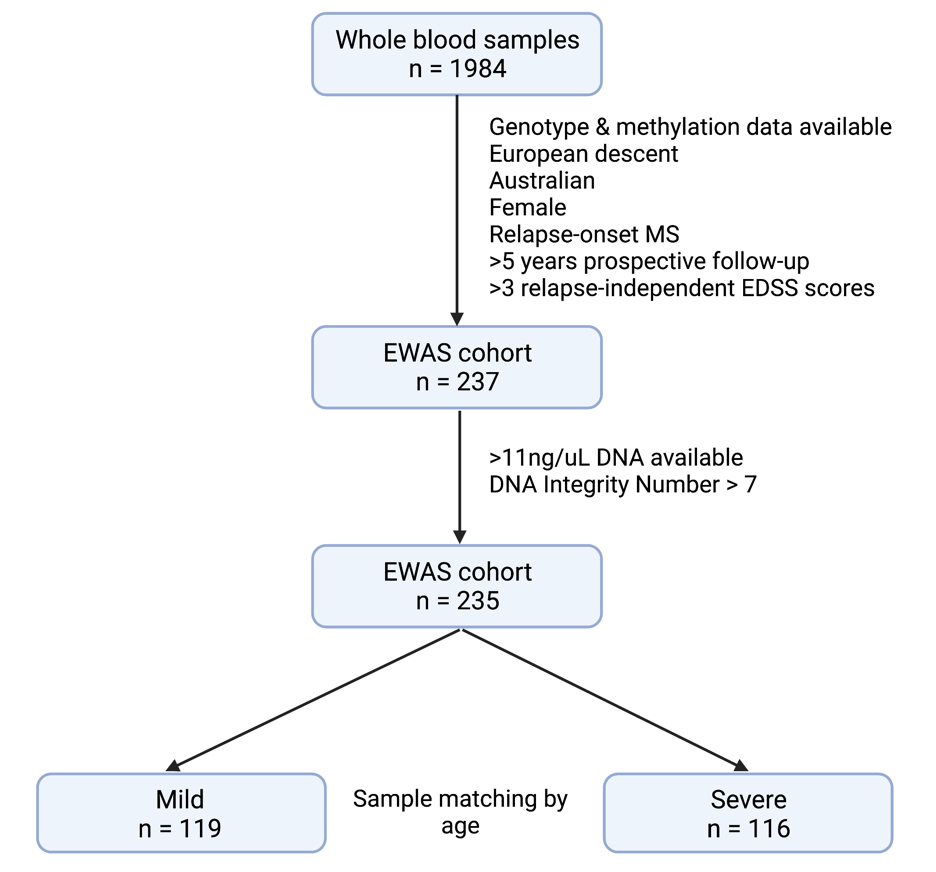


**Supplementary Figure 1.** **Participant inclusion flowchart and inclusion criteria.**

*Abbreviations: MSGP = Multiple Sclerosis Genotype Phenotype Study, EDSS = Expanded Disability Status Scale, EWAS = Epigenome Wide Association Study, DNA = Deoxyribonucleic Acid*

*Made with Biorender.com*

| **a**  **** | **b ** |
| --- | --- |
| **c**  **** | **d**  **** |

**Supplementary Figure 2.** **Quality control plots produced in ChAMP.** **a)** Multidimensional Scaling (MDS) Plot of methylation beta values for all samples, with the 1000 most variable positions displayed. There is no grouping of mild or severe groups evident **b)** A density plot of raw methylation data prior to normalisation, plotting the beta value distribution for all samples. **c)** MDS plot after beta value normalisation. **d)** Density plot after beta value normalisation.

*Abbreviations: MDS = Multidimensional Scaling*

| **a**  **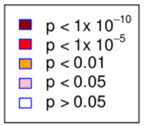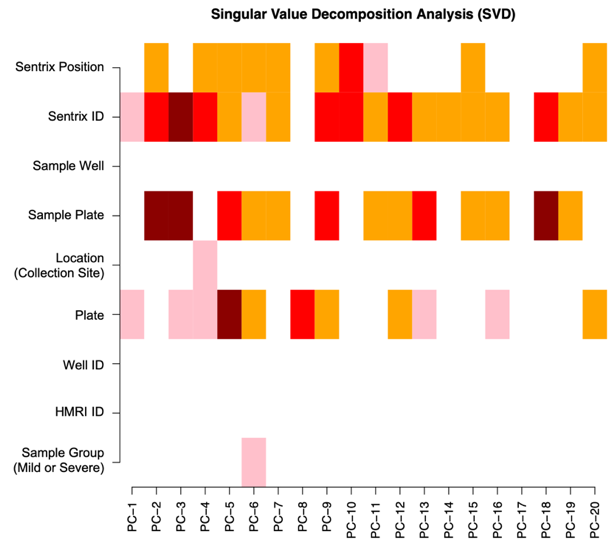** | **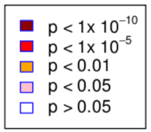b**  **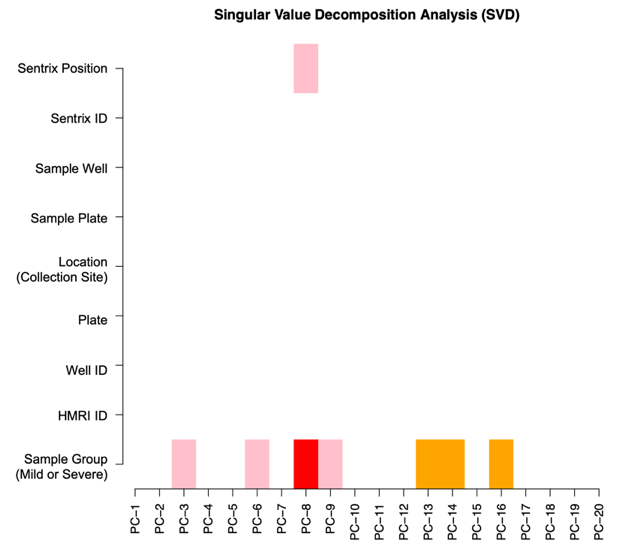** |
| --- | --- |

**Supplementary Figure 3. Methylation data batch effect correction.** Singular Value Decomposition (SVD) heatmaps before and after the removal of technical variation (batch effects) with ComBat. **a)** Before the removal of technical variation, Plate, Sentrix ID and Sentrix Position explained most of the variance in the data. **b)** Once technical variation at Plate, Sentrix ID and Sentrix Position were removed with ComBat, Sample Group explained most of the variance in the data, as expected as the variable of interest.

*Abbreviations: HMRI = Hunter Medical Research Institute, PC = Principal Component*

| **a**  **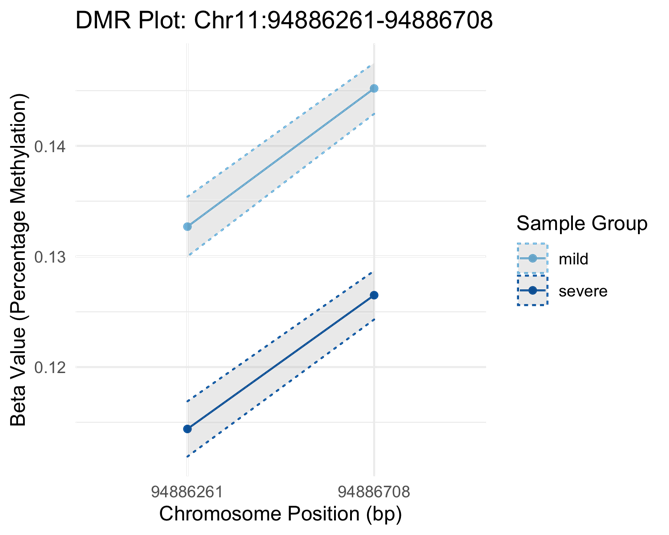** | **b**  **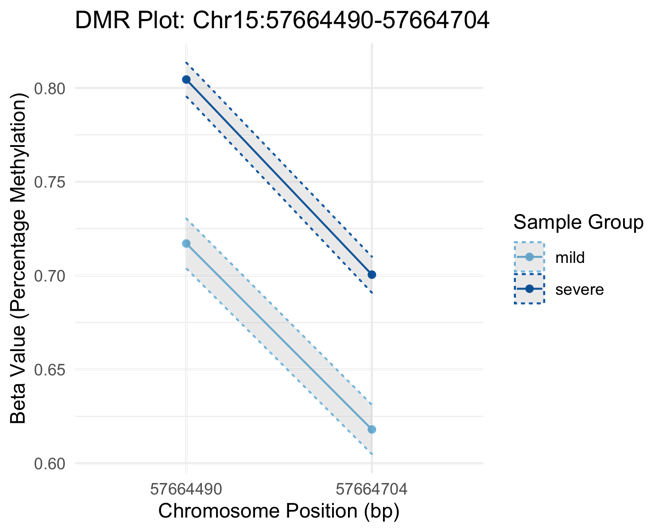** |
| --- | --- |

**Supplementary Figure 4.** **Differentially methylated regions (DMRs).** Mean methylation (β) values for mild (light blue) and severe (dark blue) patients at each CpG in **a)** DMR^Chr11^: Chr11:94886261-94886708 and **b)** DMR^Chr15^: Chr15:57664490-57664704. Grey shading shows standard error of the mean.

_
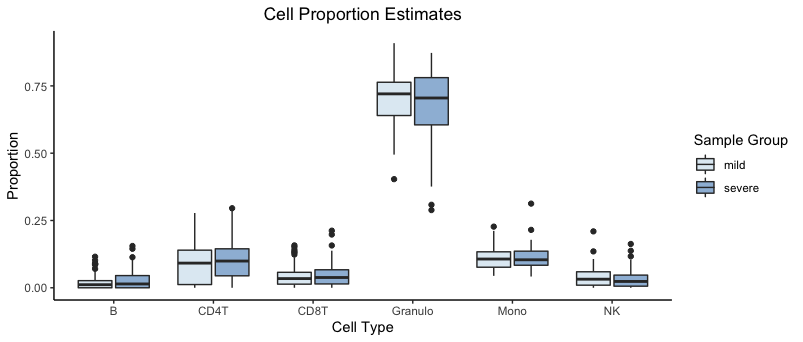
_

**Supplementary Figure 5.** **Cell type proportion estimates by disease severity.** No significant differences between mild and severe groups were found. B, p = 0.054; CD4T, p = 0.127; CD8T, p = 0.459; Granulo, p = 0.551; Mono, p = 0.671; NK, p = 0.154.

*Abbreviations: B = B cells, CD4T = CD4+ T cells, CD8T = CD8+ T cells, Granulo = Granulocytes, Mono = Monocytes, NK = Natural Killer cells*

| **a**  **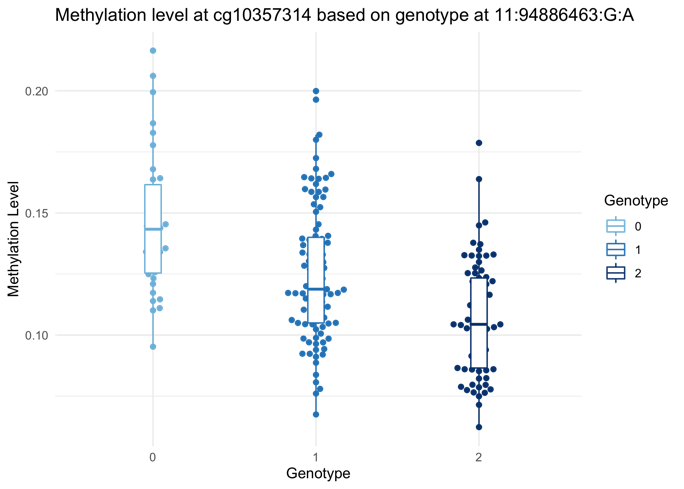** | **c**  **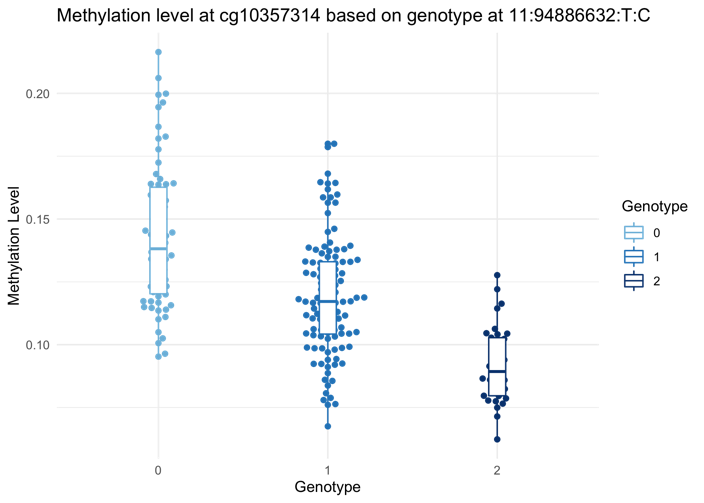** |
| --- | --- |
| **b**  **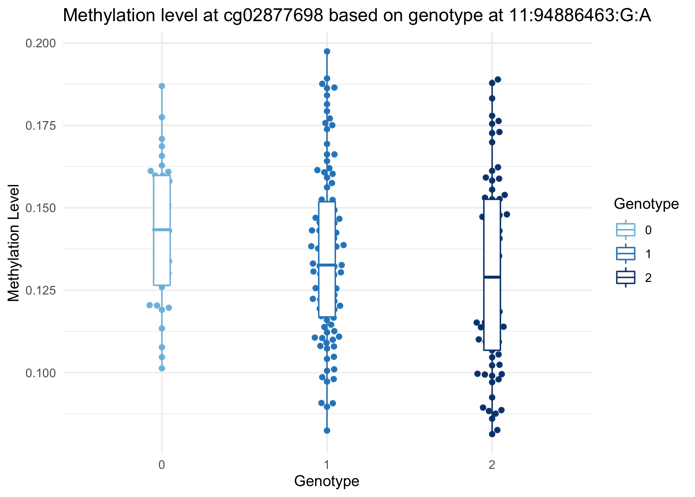** | **d**  **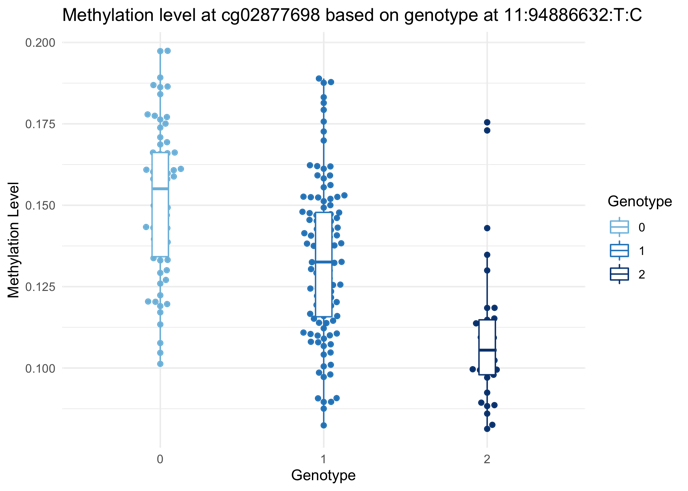** |

**Supplementary Figure 6. Methylation quantitative trait loci effect at DMR^Chr11^.** Mean methylation level at cg10357314 and cg02877698 in DMR^Chr11^ based on genotype at 11:94886463:G:A and 11:94886632:T:C. **a)** cg10357314 and 11:94886463:G:A, p = 4.69x10^-10^, **b)** cg10357314 and 11:94886632:T:C, p = 2.92x10^-16^, **c)** cg02877698 and 11:94886463:G:A, p = 0.05, **d)** cg02877698 and 11:94886632:T:C, p = 5.70x10^-13^.

| **a**  **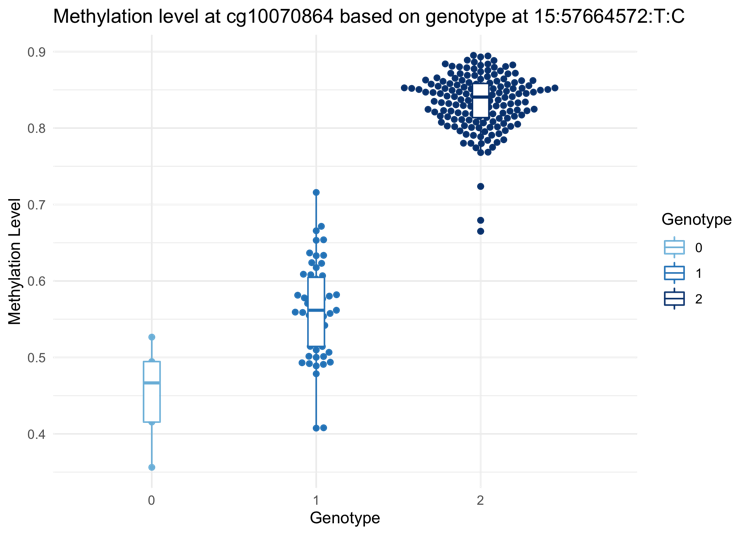** |
| --- |
| **b**  **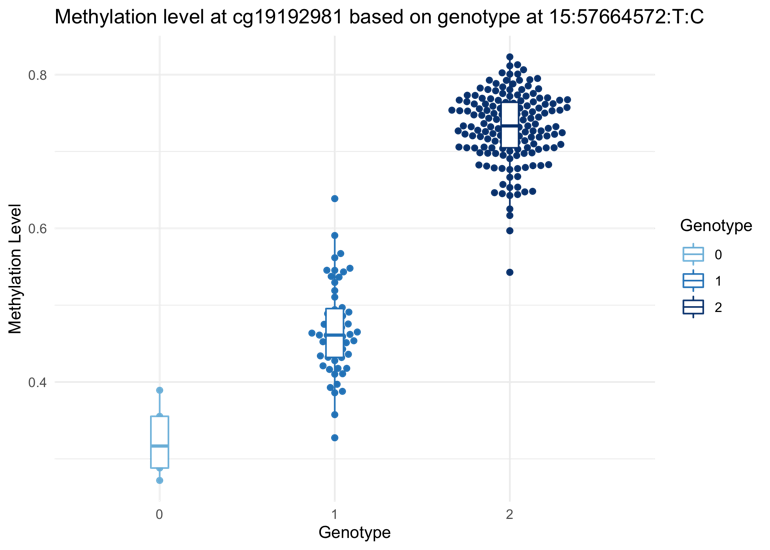** |

**Supplementary Figure 7. Methylation quantitative trait loci effect at DMR^Chr15^.** Mean methylation level at cg10070864 and cg19192981 in DMR^Chr15^ based on genotype at 15:57664572:T:C: **a)** cg10070864, p = 3.12x10^-07^, **b)** cg19192981, p = 9.93x10^-06^.

**
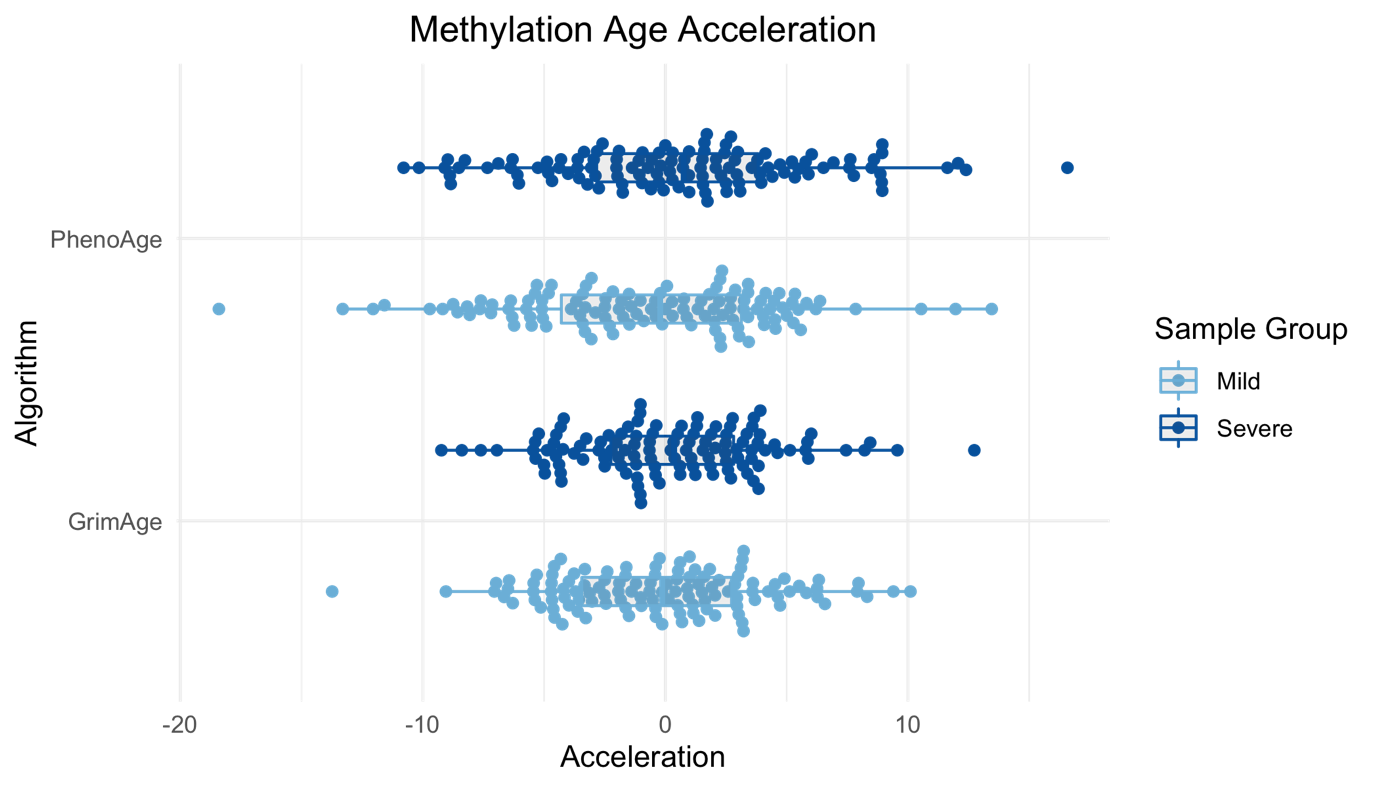
**

**Supplementary Figure 8. Methylation age acceleration (MAA) between mild and severe groups.** MAA was calculated using PhenoAge and GrimAge algorithms. There were significant differences in MAA between mild and severe groups using PhenoAge (Δμ = 1.36, p=0.048), but not GrimAge (Δμ = 0.464, p = 0.375).
